# Comprehensive prognostic model for immunotherapy in small cell lung cancer: a multi-center study integrating clinical and blood biomarkers

**DOI:** 10.3389/fonc.2025.1680624

**Published:** 2025-09-10

**Authors:** Qiuqiao Mu, Yuhao Jing, Yun Ding, Jingxian Wang, Han Zhang, Yuhang Jiang, Lin Tan, Jie Zhang, Xin Li, Daqiang Sun

**Affiliations:** ^1^ Clinical School of Thoracic, Tianjin Medical University, Tianjin, China; ^2^ Department of Thoracic Surgery, Tianjin Chest Hospital, Tianjin, China; ^3^ Chest Hospital, Tianjin University, Tianjin, China; ^4^ Department of Thoracic Surgery, Fujian Provincial Hospital Affiliated to Fuzhou University, Fuzhou, Fujian, China; ^5^ Department of Cardiology, Yantai Yuhuangding Hospital, Yantai, China; ^6^ Qingdao Hospital, University of Health and Rehabilitation Sciences (Qingdao Municipal Hospital), Qingdao, China

**Keywords:** small cell lung cancer, multi-center study, prognostic model, blood biomarkers, immunotherapy

## Abstract

**Background:**

Small cell lung cancer (SCLC) is a highly aggressive and rapidly progressing form of lung cancer that is difficult to treat. Immunotherapy has provided encouraging outcomes, but only a small proportion of patients experience significant benefit. Predicting which patients will respond to immunotherapy is essential for maximizing treatment effectiveness.

**Methods:**

This retrospective analysis included 319 SCLC patients from multiple centers in China who underwent immune checkpoint inhibitor (ICI) therapy. Clinical features and peripheral blood biomarkers were used together to create a prediction system. This system aims to forecast overall survival (OS) and progression-free survival (PFS). Univariate and multivariate Cox regression analyses were used to identify prognostic factors. A nomogram was then constructed to perform risk stratification. The model’s performance was evaluated using multiple methods. Time-dependent ROC analysis was applied to assess its predictive accuracy. Decision curve analysis (DCA) was used to determine its clinical utility. Additionally, calibration plots were created to examine the model’s consistency with actual outcomes.

**Results:**

In SCLC patients, age, brain metastasis, cigarettes per day, lnNSE (Natural Logarithm of Neuron-Specific Enolase), lnAISI (Natural Logarithm of the Aggregate Immune-Inflammatory Index), and lnCLR (Natural Logarithm of the CRP-to-Albumin Ratio) were found to be key factors affecting OS. A nomogram incorporating six variables exhibited excellent discrimination, calibration, and practical utility in both training and validation cohorts. Notably, lnAISI and lnCLR, indicators of systemic immune-inflammation, showed significant predictive value.

**Conclusion:**

This study developed a convenient and effective multi-factor survival prediction model based on clinical and hematological markers. The model provides a tool for personalized management of immunotherapy in SCLC patients. It offers new insights and practical evidence for precision treatment in SCLC.

## 1 Introduction

Lung cancer is the leading cause of death related to cancer globally. Each year, approximately 1.8 million individuals are diagnosed, and about 1.6 million lose their lives to this disease (1). Lung cancer is primarily categorized into two types: non-small cell lung cancer (NSCLC), comprising 80-85% of cases, and SCLC, accounting for 10-15% (2). It rapidly expands, disseminates early, and is associated with a poor prognosis. The five-year survival rate ranges from 12% to 30% (3–6). The choice of treatment depends on the stage. Common options include surgery, chemotherapy, radiotherapy, and immunotherapy (6).

In recent years, immunotherapy for SCLC has gained increasing attention. SCLC is an aggressive form of lung cancer characterized by rapid growth and a poor prognosis. For many years, treatment choices were limited. Combining chemotherapy with immune checkpoint inhibitors, particularly anti-PD-L1 monoclonal antibodies, has led to better survival outcomes in patients with ES-SCLC. This represents a significant shift in treatment. The pivotal IMpower133 trial revealed that incorporating the anti-PD-L1 monoclonal antibody atezolizumab into standard chemotherapy (carboplatin + etoposide) notably extended overall survival from a median of 10.3 months to 12.3 months, thereby setting immune combination chemotherapy as the new first-line standard for ES-SCLC (7). The CASPIAN study later supported this finding, showing that the combination of durvalumab, an anti-PD-L1 drug, with platinum-based chemotherapy and etoposide also improved survival (8). However, the effect of immunotherapy is still limited to a smaller group of patients (9–11). It is crucial to identify SCLC patients who may respond to ICIs and to discover predictive biomarkers for ICI treatment (12).

Unlike in NSCLC, tissue biomarkers commonly used in SCLC, such as PD-L1 expression and TMB, do not consistently provide reliable predictive value. A meta-analysis of 27 studies involving 2792 SCLC patients found that PD-L1 expression in tumor tissue was approximately 22%–26%, with no significant correlation to overall survival (HR = 0.86, 95% CI 0.49–1.50, p = 0.588). Another study indicated that PD-L1 expression was not correlated with clinical staging, LDH levels, or various other indicators (13). Although TMB has been explored in SCLC, data is limited and testing is inconsistent, so there is still no clinical guidance. In this context, blood inflammation markers like SIRI, AISI, NLR, PLR, and LDH have gained attention due to their easy detection and dynamic monitoring advantages (14–19). Numerous meta-analyses and multicenter studies demonstrate a strong association between elevated NLR and reduced PFS and OS in SCLC patients, while the predictive significance of PLR is variable. Furthermore, both LDH and PLR exhibit potential as predictive markers. Several studies have found that high LDH levels are related to OS in SCLC patients (20). However, current studies often focus on single inflammation markers or clinical variables and lack integrated prediction models that combine multiple blood biomarkers with clinical features. There has also been limited systematic evaluation of their clinical applicability, which restricts their use in precision treatment. Therefore, it is urgent to explore multi-factor prediction tools that integrate clinical features and blood biomarkers to optimize immune treatment risk stratification and dynamic efficacy monitoring in SCLC patients.

This study analyzed data from 319 SCLC patients treated with immune checkpoint inhibitors across various centers in China. We combined patients’ baseline clinical features with peripheral blood biomarkers to build a prognostic model for OS and PFS in patients receiving immunotherapy. This model can guide early risk stratification and help optimize treatment, supporting the shift of SCLC care from empirical therapy to precision medicine.

## 2 Methods

### 2.1 Participant group

Data for this study were retrospectively collected from SCLC cases diagnosed between January 1, 2019, and April 1, 2025, from four centers in China: Tianjin Chest Hospital, Fujian Provincial Hospital, and Qingdao Municipal Hospital, Tianjin Fourth Central Hospital. Patients were eligible if they were 18 years or older, had pathologically or cytologically confirmed SCLC, and had an ECOG performance status ranging from 0 to 1. Additionally, they must have received at least one dose of an immune checkpoint inhibitor. The study included 319 patients.

To ensure reliable and interpretable results, we excluded patients with: (1) other active cancers affecting survival, (2) active infections, (3) liver, kidney, or bone marrow dysfunction, or (4) incomplete follow-up data, including missing survival status or time.

### 2.2 Data acquisition and study endpoints

Demographic and clinical baseline data were gathered for each enrolled patient. Factors considered were age, sex, smoking history, and prevalent comorbidities like hypertension, coronary heart disease, and diabetes. Tumor characteristics and the extent of disease at the first diagnosis were also recorded. We paid particular attention to distant metastases, including those in the brain, bone, and liver. Brain metastases were confirmed by three senior physicians using the patient’s medical history, brain MRI, or head CT. Bone metastases were identified with a combination of PET-CT and radionuclide bone scans. Liver metastases were evaluated using abdominal CT imaging.

Baseline peripheral blood tests were taken before treatment started. Hematologic markers assessed included neuron-specific enolase (NSE), LDH, carcinoembryonic antigen (CEA), albumin, and counts of neutrophils, lymphocytes, and monocytes. Additionally, C-reactive protein (CRP, mg/L) levels and calculated inflammatory indices were also measured. The aggregate immune-inflammatory index (AISI) was defined as:

AISI = neutrophil count × platelet count × monocyte count/lymphocyte count.

The CRP-to-albumin ratio (CLR) was calculated as: CLR = C-reactive protein (CRP) level/albumin level.

Missing variables were not excluded. Instead, we used multiple imputation to handle missing data and reduce bias. Specifically, in the training cohort, CRP had 11 missing values (4.64%), LDH had 7 missing values (2.95%), and no other variables had missing data. Since the proportion of missing data in our study was less than 5%, no sensitivity analysis was performed. The main outcomes assessed were OS and PFS.

### 2.3 Model development and evaluation

Patients from Tianjin Chest Hospital were used as the training cohort. Patients from Fujian Provincial Hospital, Tianjin Fourth Central Hospital, and Qingdao Municipal Hospital formed the external validation cohort. The primary outcomes of the study were survival status and duration.

We first performed univariate Cox proportional hazards regression on all clinical and laboratory variables. Variables with a p-value less than 0.1 were retained for multivariate analysis to ensure potential predictors were not prematurely excluded. A prognostic model was developed using multivariate Cox regression, selecting final variables based on statistical significance and clinical judgment. To account for multiple testing, p-values were adjusted using the Benjamini-Hochberg (BH) method for false discovery rate (FDR) control. A nomogram was developed to visually represent the model, whose discrimination, calibration, and clinical utility were assessed in both the training and validation cohorts. We performed time-dependent ROC analysis to assess the model’s predictive accuracy over time. Calibration plots were generated to examine how well the predicted survival probabilities matched the actual outcomes. Additionally, decision curve analysis (DCA) was used to evaluate the clinical utility of the model in predicting overall survival at both 12 and 24 months. Risk stratification was carried out with X-tile software using risk scores from the nomogram. Patients were categorized into high-risk and low-risk groups. Kaplan–Meier curves were used to compare the survival outcomes between the two groups, with the log-rank test applied for significance. Additionally, we conducted subgroup survival analysis to assess the stability of the risk model in patients with ES-SCLC and LS-SCLC disease. Finally, to compare the model’s discrimination with that of individual biomarkers, we calculated time-dependent AUC values for the integrated model and for three single indicators: lnNSE, lnAISI, and lnCLR.

### 2.4 Statistical analysis

Categorical variables were presented as frequencies and proportions. For comparisons between groups, the Mann–Whitney U test was utilized for continuous data, while either the chi-square test or Fisher’s exact test was applied for categorical data, depending on the context. All statistical analyses and visualizations were conducted using RStudio (version 4.2.1).

## 3 Results

### 3.1 Initial characteristics

The training cohort comprised 237 patients, and the validation cohort included 82 patients. In the training cohort, the median OS was 12.0 months (IQR: 6.0–19.1), while the median PFS was 8.73 months (IQR: 4.77–16.17). In the validation cohort, the median OS was 12.62 months (IQR: 7.0–18.45), and the median PFS was 10.18 months (IQR: 4.75–14.38). The two sets showed no significant differences in demographic characteristics, disease stage, comorbidities, laboratory results, or survival outcomes. The similarities show that the two datasets are suitable for further examination (**Table 1**).

**Table 1:** Baseline clinical characteristics of small cell lung cancer patients in the training and external validation cohorts.

Variables	Total (N=319)	Training cohort (N=237)	Validation cohort (N=82)	P value
Age, year, median (IQR)	66 (60, 71)	66 (60, 71)	66 (59.25, 70)	0.466
Gender, n (%)				0.912
Female	59 (18.5)	43 (18.14)	16 (19.51)	
Male	260 (81.5)	194 (81.86)	66 (80.49)	
VALG, n (%)				0.166
LS−SCLC	156 (48.9)	110 (46.41)	46 (56.1)	
ES−SCLC	163 (51.1)	127 (53.59)	36 (43.9)	
Brain metastasis, n (%)	40 (12.54)	28 (11.81)	12 (14.63)	0.638
Bone metastasis, n (%)	72 (22.57)	59 (24.89)	13 (15.85)	0.125
Liver metastasis, n (%)	29 (9.09)	21 (8.86)	8 (9.76)	0.984
Smoking years, median (IQR)	40 (20,40)	40 (20, 40)	32.5 (10, 40)	0.075
Cigarettes per day, median (IQR)	20 (5, 20)	20 (10, 20)	20 (0, 20)	0.48
Quit smoking, n (%)	55 (17.24)	41 (17.3)	14 (17.07)	1
Hypertension, n (%)	131 (41.07)	100 (42.19)	31 (37.8)	0.571
CHD, n (%)	44 (13.79)	34 (14.35)	10 (12.2)	0.763
Diabetes, n (%)	66 (20.69)	50 (21.1)	16 (19.51)	0.883
NSE, ng/ml, median (IQR)	21.6 (14.15, 53.4)	21.4 (15, 41.9)	21.66 (11.94, 212.18)	0.739
LDH, U/L, median (IQR)	212 (177.5, 268.5)	212 (178, 276)	210 (173.25, 247.75)	0.241
CEA, ng/ml, median (IQR)	3.35 (2.23, 6.58)	3.61 (2.22, 6.68)	3.02 (2.26, 5.33)	0.368
WBC,10^9/L, median (IQR)	7.06 (5.89, 8.65)	7.29 (5.95, 8.58)	6.65 (5.39, 8.96)	0.397
Neutrophils, 10^9/L, median (IQR)	4.42 (3.31, 5.95)	4.52 (3.49, 5.8)	4.1 (3, 6.28)	0.233
Lymphocytes, 10^9/L, median (IQR)	1.71 (1.34, 2.1)	1.7 (1.39, 2.07)	1.75 (1.28, 2.2)	0.858
Monocyte, 10^9/L, median (IQR)	0.45 (0.36, 0.62)	0.46 (0.36, 0.61)	0.44 (0.33, 0.64)	0.737
Albumin, g/L, median (IQR)	40.5 (38.3, 42.85)	40.3 (38, 42.6)	41.15 (39, 43.32)	0.07
CRP, mg/L, median (IQR)	12.1 (4.85, 35)	12.8 (5.05, 33.3)	10.5 (3.93, 43.5)	0.867
PNI, Mean ± SD	49.44 ± 5.34	49.27 ± 5.17	49.94 ± 5.79	0.353
AISI, median (IQR)	323.26 (175.71, 625.33)	332.24 (178.29, 616.53)	305.75 (159.58, 676.18)	0.723
CLR, median (IQR)	7.11 (2.58, 23.2)	7.16 (2.8, 21.13)	6.1 (2.03, 29.78)	0.891
lnNSE, median (IQR)	3.07 (2.65, 3.98)	3.06 (2.71, 3.74)	3.08 (2.48, 5.36)	0.736
lnAISI, Mean ± SD	5.75 ± 0.92	5.76 ± 0.85	5.73 ± 1.09	0.784
lnCLR, median (IQR)	1.96 (0.95, 3.14)	1.97 (1.03, 3.05)	1.81 (0.71, 3.39)	0.891
PFS status, n (%)	249 (78.06)	185 (78.06)	64 (78.05)	1
PFS months, median (IQR)	9 (4.73, 15.18)	8.73 (4.77, 16.17)	10.18 (4.75, 14.38)	0.710
OS status, n (%)	224 (70.22)	168 (70.89)	56 (68.29)	0.762
OS months, median (IQR)	12.27 (6.06, 18.95)	12 (6, 19.1)	12.62 (7, 18.45)	0.542

VALG, Veterans Administration Lung Cancer Study Group; LS-SCLC, Limited-Stage Small Cell Lung Cancer; ES-SCLC, Extensive-Stage Small Cell Lung Cancer; CHD, Coronary Heart Disease; NSE, Neuron-Specific Enolase; LDH, Lactate Dehydrogenase; CEA, Carcinoembryonic Antigen; WBC, White Blood Cell count; CRP, C-reactive Protein; PNI, Prognostic Nutritional Index; AISI, Aggregate Index of Systemic Inflammation; CLR, C-reactive Protein-to-Lymphocyte Ratio; PFS, Progression-Free Survival; OS, Overall Survival.

Clinical characteristics at baseline for SCLC patients in the training cohort, stratified by survival status, are summarized in **Supplementary Table S1**. Compared with survivors, patients who died were older (p = 0.017) and more often male (p = 0.026). They also had higher levels of serum NSE, LDH, CRP, and inflammation-related indices (AISI and CLR) as well as lower lymphocyte counts. These results suggest that systemic inflammation and tumor burden are closely related to worse prognosis.

### 3.2 Cox regression analysis for single and multiple variables

**Figure 1:**
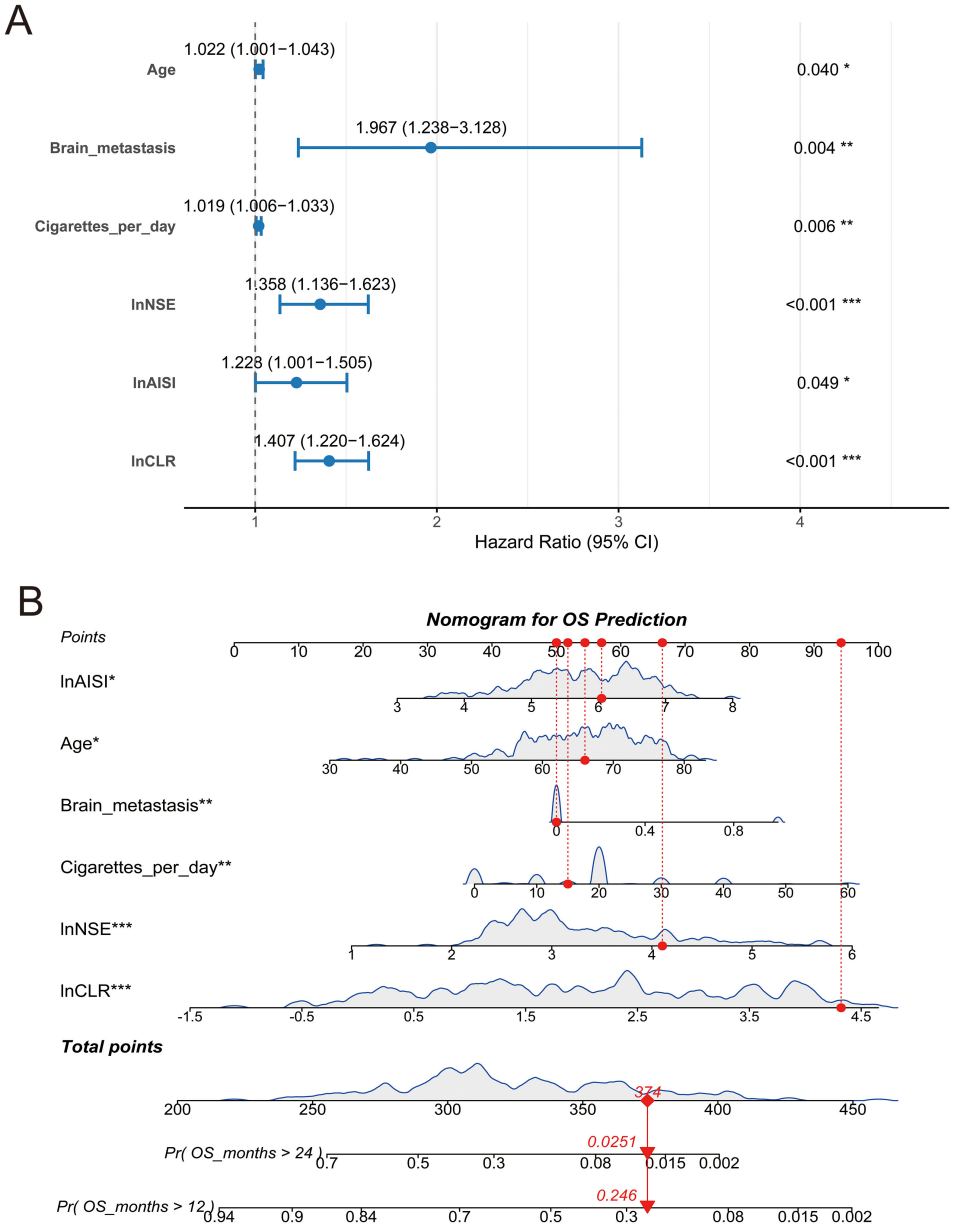
Forest plot and nomogram for overall survival prediction. **(A)** Multivariate Cox regression forest plot showing hazard ratios (HR) and 95% confidence intervals (CI) for prognostic variables; **(B)** Nomogram based on the final Cox model to predict the probabilities of 12-month and 24-month overall survival. Red lines indicate an example patient’s point allocations and predicted survival probabilities. *p < 0.05, **p < 0.01, and ***p < 0.001.

**Table 2** displays the univariate and multivariate Cox regression analysis results for overall survival in small cell lung cancer patients. Univariate analysis identified several variables associated with patient prognosis, such as age (p < 0.001), VALG (p = 0.008) brain metastases (p = 0.002) and liver metastases (p =0.016), cigarettes per day (p = 0.004), and the logarithmic values of NSE (p < 0.001), LDH (p < 0.001), AISI (p < 0.001), and CLR (p < 0.001). To ensure that potential predictive factors are not prematurely excluded, we retained variables with a p-value less than 0.1 for multivariate analysis. The multivariate analysis demonstrated that age (HR: 1.022, 95% CI: 1.001-1.043, p = 0.040), brain metastasis (HR: 1.967, 95%CI: 1.238-3.128, p = 0.004), Cigarettes per day (HR: 1.019, 95%CI: 1.006-1.033, p = 0.006), lnAISI (HR: 1.228, 95%CI: 1.001-1.505, p = 0.049), lnCLR (HR: 1.407, 95%CI: 1.220-1.624, p < 0.001), and lnNSE (HR: 1.358, 95%CI: 1.136-1.623, p = 0.001) were independent indicators of worse survival outcomes, even after the Benjamini-Hochberg (BH) multiple testing correction (**Figure 1A**, **Table 2**). Spearman correlation analysis showed weak positive correlations between Age and Brain metastasis (r = 0.13, p = 0.049), Age and lnCLR (r = 0.13, p = 0.049), Brain metastasis and lnNSE (r = 0.14, p = 0.028), lnNSE and lnCLR (r = 0.20, p = 0.002), and a moderate positive correlation between lnAISI and lnCLR (r = 0.53, p < 0.001). The VIF values for Age, Cigarettes per day, Brain metastasis, lnNSE, lnAISI, and lnCLR were 1.078, 1.063, 1.008, 1.213, 1.240, and 1.053, respectively, indicating no collinearity between these variables and other independent variables (**Supplementary Figure S3**).

**Table 2:** Results of univariate and multivariate Cox regression analyses for overall survival.

Variables	Univariate COX regression		Multivariate COX regression		
	HR (95% CI)	P value	HR (95% CI)	P value (unadjusted)	P value (BH adjusted)
Age	1.039 (1.019–1.060)	<0.001	1.022(1.001-1.043)	0.040	0.048
Gender					
Female	Reference	–	Reference	–	–
Male	1.400 (0.908–2.160)	0.128	0.950 (0.592-1.332)	0.827	0.886
VALG					
LS−SCLC	Reference	–	Reference	–	–
ES−SCLC	1.522 (1.117–2.074)	0.008	1.029 (0.766-1.345)	0.886	0.886
Brain metastasis	2.047 (1.310–3.198)	0.002	1.967(1.238-3.128)	0.004	0.008
Bone metastasis	1.367 (0.973–1.921)	0.072	1.167 (0.81-1.673)	0.444	0.525
Liver metastasis	1.858 (1.120–3.082)	0.016	1.554 (0.992-2.217)	0.112	0.183
Smoking years	1.009 (1.000–1.018)	0.062	0.991 (0.979-1.003)	0.153	0.221
Cigarettes per day	1.018 (1.006–1.031)	0.004	1.019(1.006-1.033)	0.006	0.003
Quit smoking	1.009 (0.682–1.491)	0.965			
Hypertension	0.713 (0.522–0.974)	0.034	0.716 (0.532-1.03)	0.052	0.115
CHD	1.072 (0.700–1.642)	0.749			
Diabetes	0.983 (0.679–1.423)	0.926			
CEA	1.002 (0.999–1.004)	0.231			
lnNSE	1.512 (1.270–1.801)	<0.001	1.358(1.136-1.623)	0.001	0.002
lnLDH	1.877 (1.385–2.545)	<0.001	1.299 (0.918-1.73)	0.185	0.240
lnAISI	1.608 (1.328–1.948)	<0.001	1.228(1.001-1.505)	0.049	0.049
lnCLR	1.514 (1.336–1.715)	<0.001	1.407(1.220-1.624)	<0.001	<0.001^*^

HR, Hazard ratio; CI, Confidence interval; VALG, Veterans Administration Lung Group stage; LS-SCLC, Limited-stage small cell lung cancer; ES-SCLC, Extensive-stage small cell lung cancer; CHD, Coronary heart disease; CEA, Carcinoembryonic antigen; NSE, Neuron-specific enolase; LDH, Lactate dehydrogenase; AISI, Aggregate index of systemic inflammation; CLR, C-reactive protein-to-lymphocyte ratio; lnNSE, lnLDH, lnAISI, lnCLR, Natural log-transformed values of the respective variables.

### 3.3 Development and assessment of the nomogram

A nomogram incorporating six independent risk factors was developed to predict 1-year and 2-year OS in SCLC patients (**Figure 1B**). This nomogram is used to predict the OS of SCLC patients based on six independent variables: lnAISI, age, brain metastasis, cigarettes per day, lnNSE, and lnCLR. Each variable’s contribution is translated into a corresponding score. Each feature has a scale, with higher scores indicating a greater contribution of the variable to poor survival. The total score is calculated by summing the individual scores of each variable, and then the total score is converted into predicted survival probabilities (e.g., OS greater than 12 months and 24 months). The figure shows the predicted probabilities for OS greater than 24 months and OS greater than 12 months, with corresponding values (e.g., the probability of OS > 24 months is 0.0251, and the probability of OS > 12 months is 0.246). This model provides an effective tool for clinicians to personalize treatment and predict the prognosis of SCLC patients. The nomogram’s predictive accuracy for OS was assessed using time-dependent ROC curves, calibration plots, and decision curve analysis in both the training and validation cohorts.

**Figure 2:**
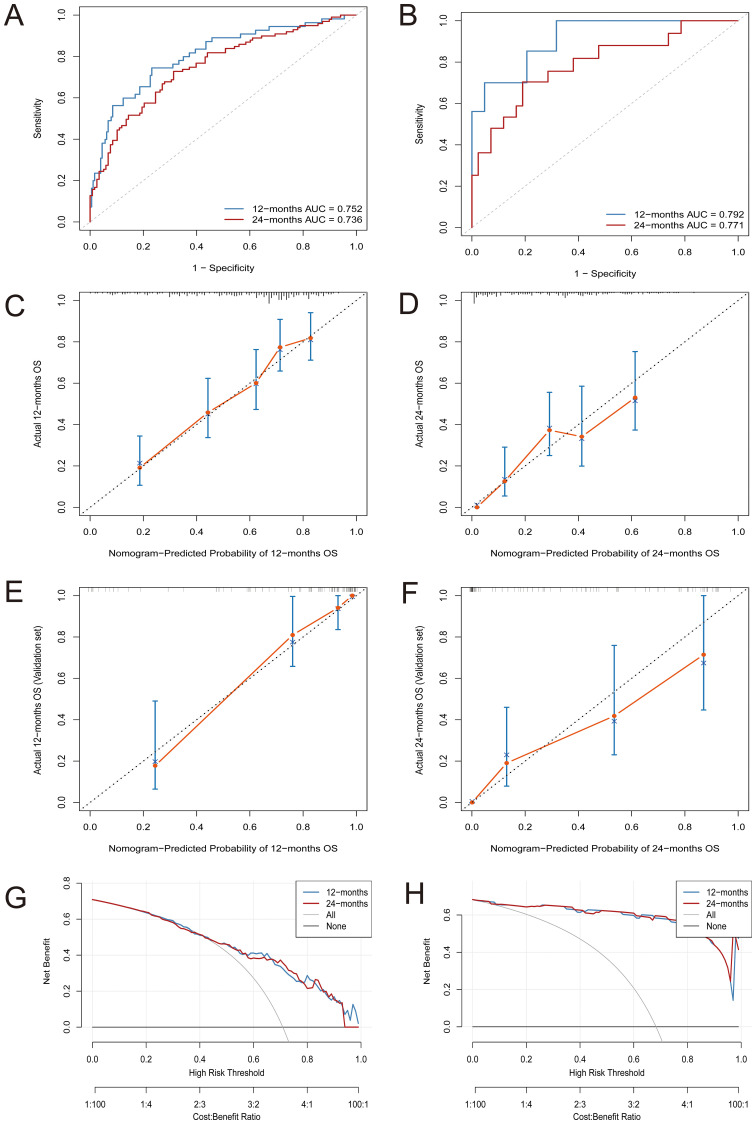
Comprehensive evaluation of the nomogram model’s predictive performance for OS. **(A)** Time-dependent ROC curves at 12 and 24 months in the training cohort; **(B)** Time-dependent ROC curves at 12 and 24 months in the validation cohort; **(C, D)** Calibration plots for predicting 12-month **(C)** and 24-month **(D)** OS in the training cohort; **(E, F)** Calibration plots for predicting 12-month **(E)** and 24-month **(F)** OS in the validation cohort; **(G, H)** Decision curve analysis (DCA) for assessing net benefit at 12 and 24 months in the training **(G)** and validation **(H)** sets.

In the training cohort, time-dependent ROC analysis revealed AUCs of 0.752 for 1-year OS and 0.736 for 2-year OS (**Figure 2A**). The model maintained stable discrimination throughout the follow-up, with AUC values consistently above 0.7 (**Supplementary Figure S1**). In the external validation cohort, the model performed slightly better, with AUCs of 0.792 and 0.771 for 1-year and 2-year OS, respectively (**Figure 2B**). Calibration analysis showed that the predicted OS closely matched the observed outcomes. In the training cohort, the calibration plots for both 1-year and 2-year survival closely followed the 45-degree reference line (**Figures 2C, D**). The external validation cohort also showed excellent calibration (**Figures 2E, F**). DCA showed that the nomogram provided greater net clinical benefit compared to treating all patients or none. The model demonstrated a consistently higher net benefit across various threshold probabilities for 1-year and 2-year overall survival in both the training and validation cohorts (**Figures 2G, H**).

### 3.4 Risk stratification and survival analysis

**Figure 3:**
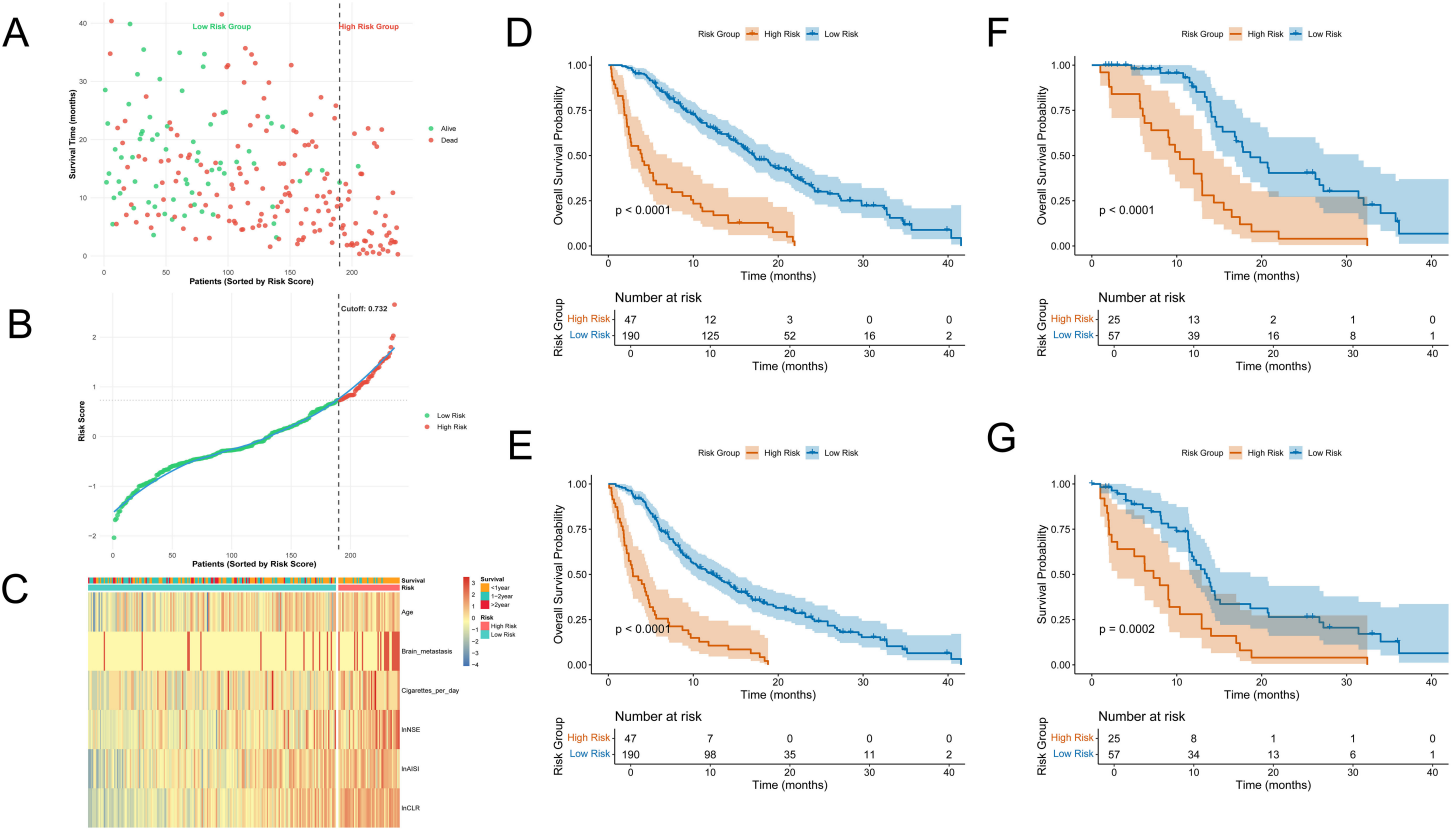
Risk stratification and survival analysis. **(A)** Survival status plot: patients are ranked by risk score, with red dots indicating death and green dots indicating survival; the y-axis represents survival time (months); **(B)** Distribution of risk scores: patients sorted by increasing risk score, with red indicating high-risk group and green indicating low-risk group; the cutoff (0.732) is indicated by the dashed line; **(C)** Heatmap of model variables across patients: rows represent the six final model variables, columns represent patients, and colors indicate relative expression or value levels; **(D, E)** Kaplan–Meier curves showing OS and PFS between high-risk and low-risk groups in the training cohort; **(F, G)** Kaplan–Meier curves showing OS and PFS between high-risk and low-risk groups in the validation cohort.

Each patient’s total risk score was calculated using the weighted coefficients of six prognostic factors from the final model. X-tile software identified an optimal cutoff value of 0.732 (**Supplementary Figure S2**), categorizing patients into low-risk and high-risk groups. Risk scores were ranked in ascending order, and a clear stratification pattern was observed (**Figure 3B**). **Figure 3A** shows the survival status of all patients. Blue dots indicate patients alive at the last follow-up, and red dots indicate those who had died. The vertical axis indicates the total survival duration. Patients classified as high-risk exhibited reduced survival rates and increased mortality.

We created a heatmap using the six model variables to explore the correlation between risk levels and variable expression (**Figure 3C**). All variables were standardized using Z-score transformation. The heatmap indicated that the high-risk group had increased lnAISI, lnCLR, and lnNSE levels, suggesting heightened systemic inflammation and tumor burden. These patients were older, had a higher smoking rate, and exhibited an increased incidence of brain metastases. In contrast, the low-risk group displayed more favorable biomarker and clinical profiles.

The prognostic performance of the risk assessment model was evaluated using KM survival analysis for OS and PFS in both the training and external validation cohorts. In the training cohort, the high-risk group showed significantly worse OS and PFS, with clearly distinct survival curves (**Figures 3D, E**). Log-rank tests indicated significant differences (p < 0.0001). Similar results were observed in the validation cohort. The KM curves for OS (**Figure 3F**) and PFS (**Figure 3G**) showed clear separation among the two groups, with statistically significant differences (OS: p < 0.0001; PFS: p = 0.0002).

### 3.5 Subgroup analysis

We subsequently examined the prognostic relevance of the nomogram-based risk scoring model across various clinical stages of SCLC. Survival analyses for subgroups were conducted in both the training and external validation cohorts for patients with ES-SCLC and LS-SCLC. Patients were categorized into high-risk and low-risk groups using a threshold of 0.732.

**Figure 4:**
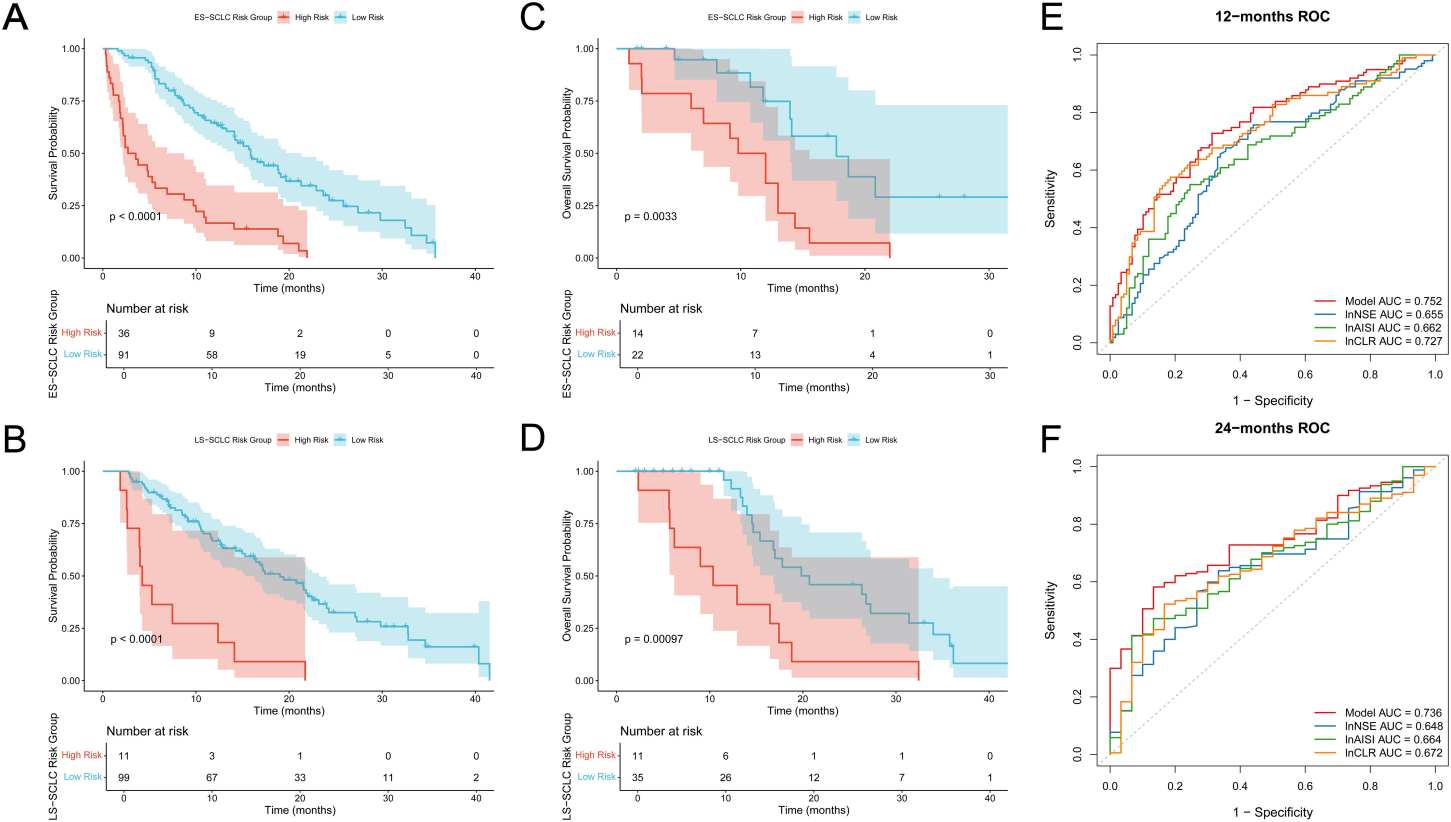
Subgroup analysis and ROC curves of individual indicators. **(A, B)** Overall survival curves for training cohort **(A)** ES-SCLC subgroup; **(B)** LS-SCLC subgroup; **(C, D)** Overall survival curves for validation cohort **(C)** ES-SCLC subgroup; **(D)** LS-SCLC subgroup; The high-risk group shows significantly worse survival outcomes than the low-risk group in both sets; **(E)** ROC curves of the integrated model and individual indicators at 12 months; **(F)** ROC curves of the integrated model and individual indicators at 24 months.

In the training cohort, KM OS curves showed clear separation between the two risk groups in both stages. **Figures 4A** (ES-SCLC) and 4B (LS-SCLC) illustrate that high-risk patients exhibited significantly reduced survival compared to low-risk patients (log-rank test, p < 0.0001).The external validation cohort showed a similar trend. In ES-SCLC, the high-risk group exhibited significantly poorer overall survival than the low-risk group (p = 0.0033, **Figure 4C**).In LS-SCLC, the survival curves were clearly distinct, showing a significant difference between the groups (p = 0.001, **Figure 4D**).

The predictive performance of the final risk model was assessed against three individual biomarkers (lnNSE, lnAISI, and lnCLR) through time-dependent ROC analysis at 12 and 24 months. **Figure 4E** indicates that the integrated model achieved a 12-month AUC of 0.752, surpassing lnNSE (0.655), lnAISI (0.662), and lnCLR (0.727).At 24 months, the model’s AUC remained 0.736, which also outperformed lnNSE (0.648), lnAISI (0.664), and lnCLR (0.672) (**Figure 4F**).These findings indicate that the integrated model provides better discriminative ability than any single biomarker.

## 4 Discussion

SCLC is an aggressive and rapidly progressing lung cancer subtype, characterized by the poorest prognosis among all types (21–23). Recently, the combination of immune checkpoint inhibitors (ICIs) with platinum-based chemotherapy has become the standard first-line treatment for ES-SCLC. Landmark trials like IMpower133 and CASPIAN have shown significant enhancements in OS and PFS; however, the OS benefit is limited, with a five-year survival rate ranging from 12% to 30% (7, 8). In clinical settings, SCLC patients exhibit a highly variable response to immunotherapy.

Some patients live longer, while most experience fast disease progression within a few months. This difference may be related to factors such as individual immune status, the tumor microenvironment, and genetic variations (24, 25). Therefore, identifying patients who will benefit most as early as possible is crucial. Developing simple and cost-effective tools for risk stratification and efficacy prediction is also necessary. These tools should be widely applicable in clinical practice. They are important steps toward achieving precision treatment in SCLC.

This study looked at 319 SCLC patients who received immunochemotherapy. We combined clinical features and blood biomarkers to create a survival prediction model. Both univariate and multivariate Cox regression were used in the analysis. Six independent predictors of overall survival were identified: age, brain metastasis, cigarettes per day, lnNSE, lnAISI, and lnCLR. Using these factors, we built a nomogram to estimate survival probabilities for 1 year and 2 years. The model showed strong discrimination, calibration, and clinical utility in both the training and external validation cohorts.

In this study, lnAISI and lnCLR were identified as independent risk factors for OS in the multivariate analysis, significantly enhancing the model. AISI reflects the overall inflammatory and immune status in the tumor microenvironment by integrating neutrophils, monocytes, platelets, and lymphocytes (26). Inflammation associated with tumors significantly contributes to cancer progression and metastasis by modifying the tumor microenvironment, enhancing angiogenesis, and facilitating immune evasion, thereby providing tumor cells with a growth advantage (27, 28). Inflammation can inhibit T-cell function and alter the microenvironment to generate pro-tumor cytokines like IL-6 and TNF-α, thereby diminishing the anti-tumor efficacy of ICIs (29, 30). CLR combines CRP, an inflammation marker, with lymphocytes, an immune indicator. High CLR levels indicate a state of “inflammatory activation and immune suppression”, which may reduce the efficacy of ICIs. Prior research has established a strong correlation between CLR and prognosis in various solid tumors (31–33). This study confirms its prognostic significance in SCLC immunotherapy.

In addition to blood biomarkers, this study found that brain metastasis and cigarettes per day significantly affected the effectiveness of immunotherapy. Patients with brain metastasis had much shorter survival, likely due to the blood-brain barrier limiting immune cell infiltration and drug delivery. Previous studies have shown that brain metastasis reduces the effectiveness of PD-1/PD-L1 inhibitors in NSCLC patients, and our study confirms this in SCLC (34). Smoking, a major cause of SCLC, may increase TMB, which enhances tumor immunogenicity. However, long-term smoking can also cause chronic lung inflammation and create an immunosuppressive environment, which may reduce the response to ICIs. These findings suggest that clinicians should consider the dual impact of smoking burden when evaluating treatment outcomes (35).

The innovation of this study lies in the first-time application of lnAISI and lnCLR as independent prognostic factors in the survival prediction model for SCLC immunotherapy. These markers sensitively reflect the degree of systemic inflammation and reveal the functional status of the immune system. Compared to single markers such as NLR or PLR, lnAISI and lnCLR, when combined with other common factors in the model, provide a more systematic and comprehensive assessment of the patient’s immune-inflammatory status. Furthermore, the study not only developed the prognostic model using the training cohort but also validated it through multi-center external cohorts, demonstrating the model’s wide applicability and reliability across different patient groups. This approach significantly enhances the clinical utility of the model, offering stable prognostic evaluations across various treatment centers and patient backgrounds, thus providing strong support for personalized immunotherapy management.

Despite its clinical value, this study has some limitations. We acknowledge the potential biases introduced by the retrospective design of this study, particularly in terms of patient selection and data collection. Since retrospective studies are inherently subject to selection bias, factors such as clinical decision-making, patient health status, and institutional treatment guidelines may influence both patient selection and data quality. These factors could potentially affect the accuracy of the model’s predictions. To mitigate this, we have made every effort to ensure that the patient cohort is representative, reducing the impact of selection bias on our findings. Furthermore, we recognize that there is variability in immune checkpoint inhibitor treatment regimens between centers, including differences in drug choice, dosage, and treatment schedules. These discrepancies can impact treatment outcomes and may affect the model’s applicability across different centers. We emphasize this limitation in the discussion and suggest that future studies should include large-scale, multi-center, prospective research to validate our model and assess its stability and broad applicability under various treatment regimens. Additionally, this study did not include molecular marker data, such as PD-L1 expression and TMB. Future research could add histological and molecular markers to create a more detailed prediction model and improve its accuracy.

## 5 Conclusion

In summary, this study developed a practical and effective survival prediction model based on routine clinical features and hematologic parameters. The study found that age, brain metastasis, cigarettes per day, lnNSE, lnAISI, and lnCLR are independent prognostic indicators for overall survival in patients with small cell lung cancer. The model showed high predictive accuracy and practical value in both the training and external validation cohorts, making it a useful tool for personalized immunotherapy management. Notably, lnAISI and lnCLR are comprehensive indicators of systemic immune-inflammation status. They reflect the balance between immune response and inflammation in the tumor microenvironment, demonstrating clear predictive value. Additionally, clinical factors like brain metastasis and smoking burden significantly affect the efficacy of immunotherapy, underlining the importance of including these factors in prognosis assessments. Overall, this study offers new evidence and practical guidance for risk stratification and treatment monitoring in SCLC patients receiving immunotherapy.

## Data Availability

The original contributions presented in the study are included in the article/**Supplementary Material**. Further inquiries can be directed to the corresponding authors.

## Supplementary material

Supplementary Figure 1 Time-dependent area under the curve (AUC) plots evaluating the prognostic performance of the survival model over time.

Supplementary Figure 2 Determination of the optimal cutoff value for the nomogram-derived risk score using X-tile analysis.

Supplementary Figure 3 Correlation heatmap and variance inflation factor (VIF) results for variables. The upper part shows the correlation heatmap, where each cell displays the correlation coefficient (r value) between two variables, with the range from -1 to 1. Blue indicates a positive correlation, red indicates a negative correlation, and white indicates almost no correlation. The asterisks next to the correlation coefficients represent statistical significance: *** indicates P < 0.001, ** indicates P < 0.01, and * indicates P < 0.05. The lower part shows the VIF results, where the y-axis represents the VIF value for each variable, used to assess the degree of multicollinearity among variables.
