# Supplementary figures and images for: Comprehensive prognostic model for immunotherapy in small cell lung cancer: a multi-center study integrating clinical and blood biomarkers
Source: Front Oncol. 2025 Sep 10;15:1680624. doi: 10.3389/fonc.2025.1680624 (PMC12457103; doi:10.3389/fonc.2025.1680624)

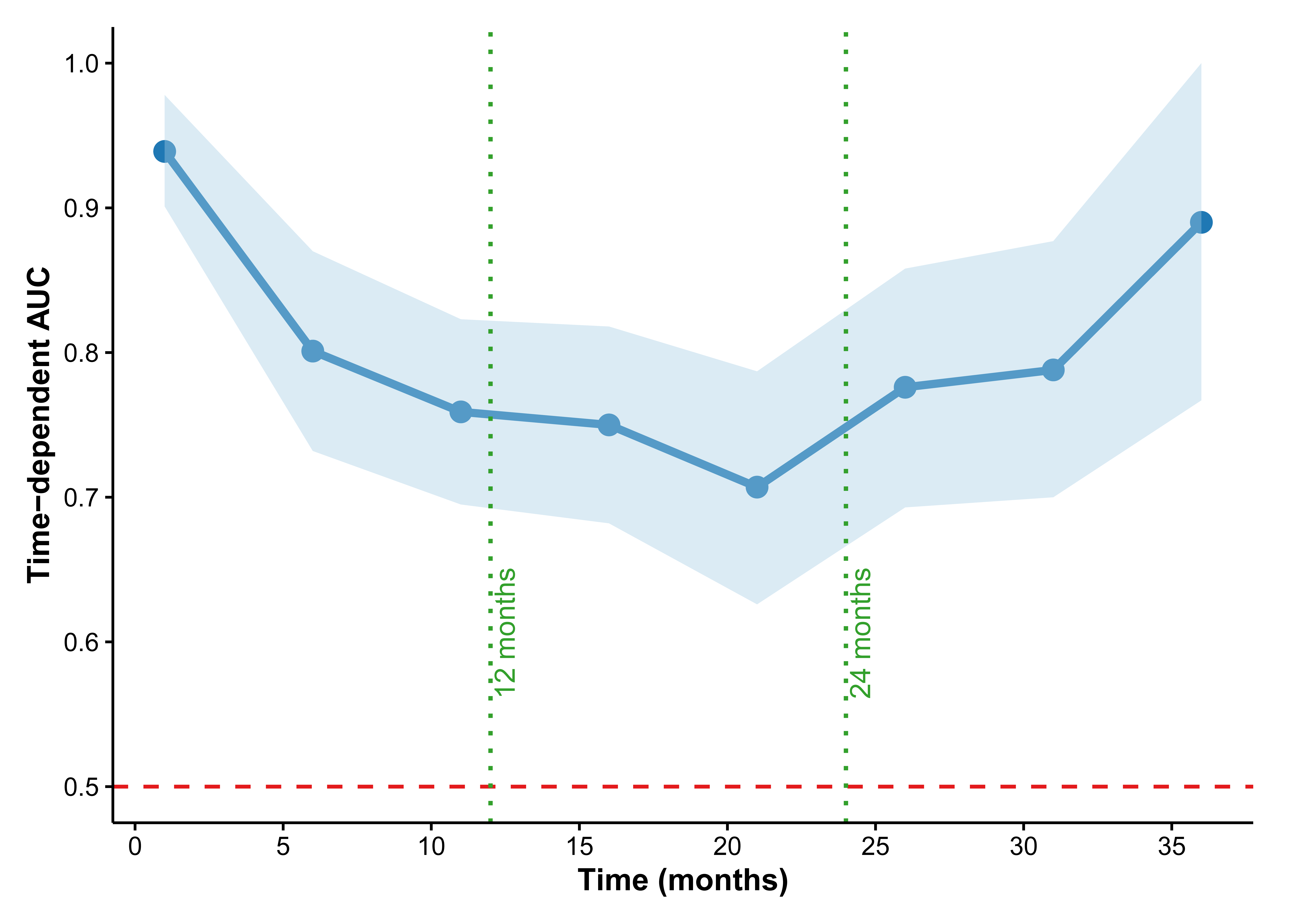

Supplement: Supplementary Figure 1 — Time-dependent area under the curve (AUC) plots evaluating the prognostic performance of the survival model over time. [file Image1.tif]

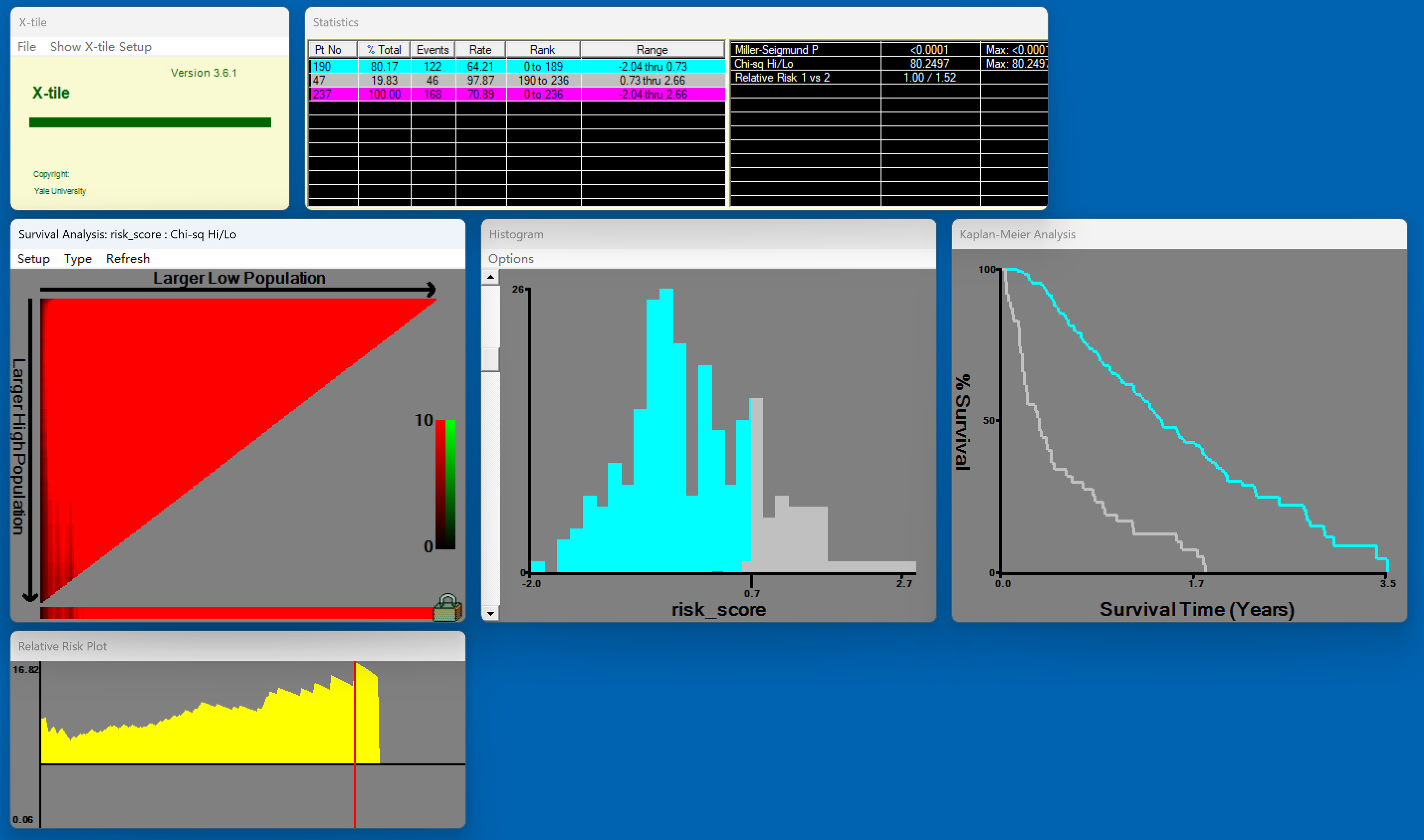

Supplement: Supplementary Figure 2 — Determination of the optimal cutoff value for the nomogram-derived risk score using X-tile analysis. [file Image2.jpeg]

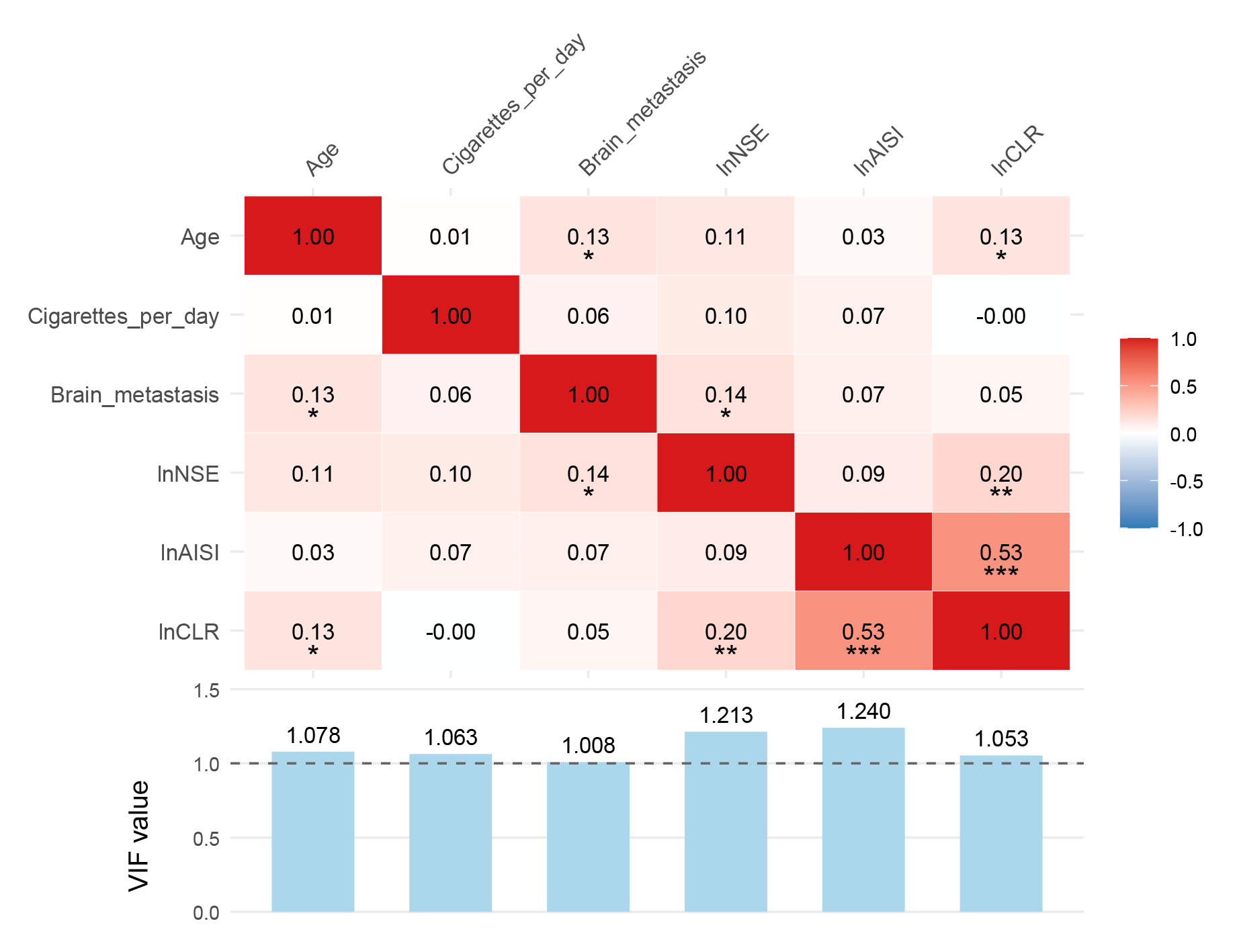

Supplement: Supplementary Figure 3 — Correlation heatmap and variance inflation factor (VIF) results for variables. The upper part shows the correlation heatmap, where each cell displays the correlation coefficient (r value) between two variables, with the range from -1 to 1. Blue indicates a positive correlation, red indicates a negative correlation, and white indicates almost no correlation. The asterisks next to the correlation coefficients represent statistical significance: *** indicates P < 0.001, ** indicates P < 0.01, and * indicates P < 0.05. The lower part shows the VIF results, where the y-axis represents the VIF value for each variable, used to assess the degree of multicollinearity among variables. [file Image3.tif]
