# Supplementary material for: Comprehensive prognostic model for immunotherapy in small cell lung cancer: a multi-center study integrating clinical and blood biomarkers
Source: Front Oncol. 2025 Sep 10;15:1680624. doi: 10.3389/fonc.2025.1680624 (PMC12457103; doi:10.3389/fonc.2025.1680624)
Supplement: Supplementary file 4 [file Table1.docx]

Table S1. Baseline clinical characteristics of small cell lung cancer patients in the training set stratified by survival status.

| Variables | Total (N = 237) | Survival (N = 69) | Death (N = 168) | *P* value |
| --- | --- | --- | --- | --- |
| Age, median (IQR) | 66 (60, 71) | 65 (58, 70) | 67 (61, 72) | 0.017 |
| Gender, n (%) |  |  |  | 0.026 |
| Female | 43 (18.14) | 19 (27.54) | 24 (14.29) |  |
| Male | 194 (81.86) | 50 (72.46) | 144 (85.71) |  |
| VALG, n (%) |  |  |  | 0.200 |
| LS−SCLC | 110 (46.41) | 37 (53.62) | 73 (43.45) |  |
| ES−SCLC | 127 (53.59) | 32 (46.38) | 95 (56.55) |  |
| Brain metastasis, n (%) | 28 (11.81) | 5 (7.25) | 23 (13.69) | 0.240 |
| Bone metastasis, n (%) | 59 (24.89) | 13 (18.84) | 46 (27.38) | 0.224 |
| Liver metastasis, n (%) | 21 (8.86) | 4 (5.8) | 17 (10.12) | 0.417 |
| Smoking years, median (IQR) | 40 (20, 40) | 30 (0, 40) | 40 (28.75, 50) | 0.058 |
| Cigarettes per day, median (IQR) | 20 (10, 20) | 10 (0, 20) | 20 (10, 20) | < 0.001 |
| Quit smoking, n (%) | 41 (17.3) | 10 (14.49) | 31 (18.45) | 0.587 |
| Hypertension, n (%) | 100 (42.19) | 35 (50.72) | 65 (38.69) | 0.119 |
| CHD, n (%) | 34 (14.35) | 9 (13.04) | 25 (14.88) | 0.871 |
| Diabetes, n (%) | 50 (21.1) | 14 (20.29) | 36 (21.43) | 0.984 |
| NSE, ng/ml, median (IQR) | 21.4 (15, 41.9) | 17 (12.4, 29.3) | 24.75 (16.33, 50.82) | < 0.001 |
| LDH, U/L, median (IQR) | 212 (178, 276) | 194 (171, 243) | 220 (182.75, 281) | 0.011 |
| CEA, ng/ml, median (IQR) | 3.61 (2.22, 6.68) | 3.64 (2.23, 6.76) | 3.58 (2.22, 6.67) | 0.953 |
| WBC,10^9/L, median (IQR) | 7.29 (5.95, 8.58) | 6.46 (5.83, 7.39) | 7.65 (6.13, 9.14) | < 0.001 |
| Neutrophils, 10^9/L, median (IQR) | 4.52 (3.49, 5.8) | 3.79 (2.85, 4.38) | 5.08 (3.84, 6.32) | < 0.001 |
| Lymphocytes, 10^9/L, median (IQR) | 1.7 (1.39, 2.07) | 1.88 (1.53, 2.42) | 1.67 (1.34, 1.98) | < 0.001 |
| Monocyte, 10^9/L, median (IQR) | 0.46 (0.36, 0.61) | 0.45 (0.34, 0.6) | 0.46 (0.37, 0.62) | 0.329 |
| Albumin, median (IQR) | 40.3 (38, 42.6) | 40.3 (37.9, 42.3) | 40.35 (38.2, 42.73) | 0.655 |
| CRP, median (IQR) | 12.8 (5.05, 33.3) | 7.71 (3.9, 12.9) | 16.7 (5.56, 47.9) | < 0.001 |
| AISI, median (IQR) | 332.24  (178.29, 616.53) | 211.04  (94.46, 371.19) | 412.75  (214.47, 704.45) | < 0.001 |
| CLR, median (IQR) | 7.16 (2.8, 21.13) | 3.95 (2.07, 8.36) | 11.18 (3.53, 32.86) | < 0.001 |
| lnNSE, median (IQR) | 3.06 (2.71, 3.74) | 2.83 (2.52, 3.38) | 3.21 (2.79, 3.93) | < 0.001 |
| lnLDH, median (IQR) | 5.36 (5.18, 5.62) | 5.27 (5.14, 5.49) | 5.39 (5.21, 5.64) | 0.011 |
| lnAISI, median (IQR) | 5.81 (5.18, 6.42) | 5.35 (4.55, 5.92) | 6.02 (5.37, 6.56) | < 0.001 |
| lnCLR, median (IQR) | 1.97 (1.03, 3.05) | 1.37 (0.73, 2.12) | 2.41 (1.26, 3.49) | < 0.001 |

Abbreviations: HR: Hazard ratio; CI: Confidence interval; VALG: Veterans Administration Lung Group stage; LS-SCLC: Limited-stage small cell lung cancer; ES-SCLC: Extensive-stage small cell lung cancer; CHD: Coronary heart disease; CEA: Carcinoembryonic antigen; NSE: Neuron-specific enolase; LDH: Lactate dehydrogenase; LDH: Lactate dehydrogenase; WBC: White blood cell count; CRP: C-reactive protein; Alb: Albumin; AISI: Aggregate index of systemic inflammation; CLR: C-reactive protein-to-albumin ratio; lnNSE, lnLDH, lnAISI, lnCLR: Natural log-transformed values of the respective variables;
